# Survey data on joint cropland management among agri-food cooperatives in Mediterranean Spanish Regions

**DOI:** 10.1016/j.dib.2022.107885

**Published:** 2022-02-02

**Authors:** Consuelo Calafat-Marzal, Francesc J. Cervera, Veronica Piñeiro, Paula Andrea Nieto-Alemán

**Affiliations:** a Group of International Economics and Development, Universitat Politècnica de València, Camino de Vera, s/n, Valencia 46022, Spain; b Departamento de Economía y Ciencias Sociales, Universitat Politècnica de València, Camino de Vera, s/n, Valencia 46022, Spain; c Departamento Agronomía, Universidad Nacional del Sur (UNS), Bahía Blanca, San Andrés 800, Bahía Blanca 8000, Argentina; d Department of Business Administration and Management, ESIC Business & Marketing School, Av. de Blasco Ibáñez, 55, Valencia 46021, Spain; e Department of Business Administration and Management, ESIC University, Cam. Valdenigriales, s/n, Pozuelo de Alarcon, Madrid 28223, Spain

**Keywords:** Joint cropland management, Agri-food cooperatives, Social innovation, Social capital, Land abandonment

## Abstract

This dataset presents data collected from joint cropland management practices survey in agri-food cooperatives of Mediterranean Spanish Regions. The objective was to examine to what extent cooperatives offer joint services, including joint management or integral exploitation of smallholdings, for the incorporation of new professionals. Data collection was conducted to five agri-food organizations: three agri-food cooperatives federations -Castilla-La Mancha, Comunitat Valenciana, and Murcia-, two second-degree agri-food cooperatives -Anecoop and Unió Nuts-, all of them located in Mediterranean Spanish Regions. A total of 1.168 survey questionnaires were distributed between July 2020 and February 2021 across five organizations through the snowball sampling method. Data from 112 collected questionnaires were correctly answered, but 106 were selected for analysis. The dataset includes socioeconomic data, productive information, and innovative characteristics from agri-food cooperatives surveyed, all in order to be able to examine the relationship between those factors and joint cropland management practices they carry on.

## Specifications Table

**Table:** 

Subject	Agricultural and Social Sciences
Specific subject area	Factors affecting joint cropland management in agri-food cooperatives from Mediterranean Spanish Regions
Type of data	Raw database and analyzed data (.xlsx) Questionnaire survey (.pdf)
How the data were acquired	Data was acquired via online survey (Google Forms), completed by CEOs of agri-food cooperatives from Mediterranean Spanish Regions. See Data Accessibility section for access to the questionnaire. Main characteristics of the data were analyzed after elimating wrong answers.
Data format	Primary, raw, and analyzed data obtained from the survey.
Description of data collection	Data were gathered through questionnaires distributed to agri-food cooperatives members of five agri-food organisations from Mediterranean Spanish Regions. The surveys were hosted in survey web platform and invitations to answer them were sent through organization networks. Data were screened for missing values and eliminating bias answers. Outliers were checked before pursuing data analysis. The final sample size consists of 106 valid responses.
Data source location	• Institution: Cooperativas Agro-alimentarias de Castilla-La Mancha, Cooperatives Agro-alimentàries de la Comunitat Valenciana, Federación de Cooperativas Agrarias de Murcia, Unió Nuts SCCL and Anecoop S. Coop. • Region: Castilla-La Mancha, Cataluña, Comunitat Valenciana and Murcia • Country: Spain
Data accessibility	Repository name: Zenodo Digital Object Identifier: 10.5281/zenodo.5511819 Direct URL to data: https://zenodo.org/record/5511819#.YZItHU6ZM2w

## Value of the Data

• The data provides agri-food cooperative information of Mediterranean Spanish Regions in terms of socioeconomic characteristics, member features, the evolution of area cultivated and land abandonment, product and governance innovation, formulas of cooperation applied, and agronomic services offered. All this information is useful to provide some guidelines to identify the conditions observed in cooperatives that implement joint cropland management initiatives.
• In addition, data can be used in studies on the level of cropland abandonment, in studies that relate the social economy to product, process and organizational innovation, and in studies on social innovation in agriculture, among others.
• The main beneficiaries of the dataset include researchers and policymakers dealing with social economy, land abandonment, and joint management initiatives. Furthermore, it is helpful to agri-food cooperatives who are coping with aiming to reorient their organizational structure to adopt different cropland management initiatives.
• The dataset can be used to identify the economic and social attributes, or combinations thereof, that characterize a cooperative profile capable of undertaking a joint cropland management strategy. Additionally, the dataset and the questionnaire elaborated may be used by other researchers who aim to conduct similar studies in other regions, either Spanish, European or other places.

## 1 Data Description

Land abandonment is currently a challenge in Europe [3], especially worrying in certain regions with a large proportion of small farms and where land fragmentation is a problem [7,11], as is the case of permanent crops of citrus orchards, vineyards and other fruits, mainly grown in the Mediterranean areas of Spain. As farms disappear without generational renewal and stop cultivating their land, many marketing cooperatives find themselves in an awkward position [9].

There are different strategies to reduce farmland/cropland abandonment [2,^4^,6]. One of them is the grouping of plots for joint cultivation. This is a recent strategy adopted by marketing cooperatives to deal with land abandonment, often due to the lack of generational renewal, which is especially useful for small-scale farming and can be considered a form of social innovation and collective entrepreneurship [10]. One significant advantage of such strategy lies in the fact that it does not necessarily change cooperatives members’ land ownership, which lowers the transaction costs of the improvement in farm structure [9].

Through this strategy, collaboration between smallholders can make it possible to efficiently address the production and management of some crops [1,^5^,8]. Joint cropland management by marketing cooperatives enables an increase in farmers’ incomes through cost reductions achieved via economies of scale and more professional management.

The dataset provides information on data collected from 106 Mediterranean-Spanish-Region agri-food cooperatives on a wide range of issues, all focused on joint cropland management practices carried on agri-food cooperatives. The survey data include the following sections: (i) socioeconomic characteristics, (ii) member features, (iii) land abandonment, (iv) product innovation, (v) governance innovations applied, (vi) inter-cooperation formulas participated in, and (vii) agronomic services and farming sections offered. The questionnaire and datasets are provided as a supplementary file.

Table 1 presents the main characteristics of the agri-food cooperatives surveyed. Most of the agri-food cooperatives are located in the region of Valencia (29%), Castellón (20%), Tarragona (17%), and Alicante (13%). They are especially dedicated to olive oil (43%), dry fruits (42%), and citrus fruits (36%). Furthermore, their average turnover in the last two years is, mainly, between 1 million € and 10 million € (47%), followed by those with a turnover from 300,000 € and 1 million € (19%), and from 10 million € to 50 million € (15%).

**Table 1:** Main characteristics of the agri-food cooperatives surveyed.

	Frequency	%
*Region*		
Alicante	14	13
Valencia	31	29
Castellón	21	20
Albacete	2	2
Ciudad Real	2	2
Cuenca	2	2
Toledo	2	2
Tarragona	18	17
Lleida	2	2
Murcia	7	7
Other	5	5

*Main type of product (multiple response)*		
Citrus fruit	38	36
Other fruits	31	29
Vine (wine)	23	22
Dry fruits	44	42
Olive grove (oil)	46	43
Vegetables	9	8
Supply	26	25
Agrarian services	9	8
Grain	6	6
Other	10	9

*Value of the cooperative's turnover (avg. last two years)*		
From 0 to 300,000 Euro	10	9
From 300,000 to 1 M Euro	20	19
From 1 M Euro to 10 M Euro	50	47
From 10 M euros to 50 M euros	16	15
More than 50M	3	3
No data	7	7

Table 2 shows member features of the agri-food cooperatives surveyed. Generally, 48% of them have between 100 and 500 members. On the one hand, most of them (53%) have a percentage between 0% and 10% of members under 40 years old. Of those cooperatives who have members under 40, 40% have a percentage more than 20% of professional members. On the other hand, 36% of the cooperatives have a ratio between 50% and 75% of members who are more than 65 years old, and 28% a percentage between 25% and 50%. Finally, 43% of agri-food cooperatives have a share of more than 75% of no-professional members, followed by 20% of those who all members are professional.

**Table 2:** Members features of the agri-food cooperatives surveyed.

	Frequency	%
*Number of members*		
< of 100	19	18
Between 100 and 500	51	48
> of 500	36	34

*Percentage of members under 40? (from 0 to 100)*		
0	10	9
Between 0 and 5	28	26
Between 5 and 10	29	27
Between 10 and 20	19	18
> of 20	19	18
No data	1	1

*Percentage of professional members under 40? (from 0 to 100)*		
0	18	19
Between 0 and 5	25	26
Between 5 and 10	6	6
Between 10 and 20	9	9
> of 20	38	40

*Percentage of members over 65? (from 0 to 100)*		
0	3	3
Between 0 and 25	20	19
Between 25 and 50	30	28
Between 50 and 75	38	36
> of 75	12	11
No data	3	3

*Percentage of no-professional members? (from 0 to 100)*		
0	21	20
Between 0 and 25	15	14
Between 25 and 50	11	10
Between 50 and 75	13	12
> of 75	46	43

The land abandoned in the agri-food cooperatives is 51% between 0% and 25% of the total land, as shown in Table 3. Furthermore, the average land abandonment rate of most agri-food cooperatives surveyed (53%) is less than 5 hectares per year. In general terms, 28% of them have not changed their cultivated area, but 57% have reduced it with different rates (less than 5%, 12%; from 5% to 10%, 21%; more than 10%, 24%). The main reasons for land abandonment are poor performance (53%) and retirements (50%).

**Table 3:** Land abandonment of the agri-food cooperatives surveyed.

	Frequency	%
*Percentage of abandoned land in the last 5 years? (from 0 to 100)*		
0	9	8
Between 0 and 25	54	51
Between 25 and 50	20	19
Between 50 and 75	7	7
> of 75	15	14
No data	1	1

*Average rate of land abandonment? (ha/year)*		
< 5 ha	56	53
5 to 10 ha	29	27
10 to 20 ha	9	8
> 20 ha	5	5
No data	7	7

*Change in terms of cultivated area over the last five years?*		
Loss of more than 10%	25	24
Loss from 5 to 10%	22	21
Loss of less than 5%	13	12
Approximately stable	30	28
Increase of less than 5%	6	6
Increase from 5% to 10%	7	7
Increase of more than 10%	3	3

*If lost, what are the reasons for this decrease? (multiple response)*		
Abandonment of plots of land due to poor performance	51	53
Abandonment of plots due to retirements	48	50
Abandonment of plots due to deaths	26	27
Plots that leave the cooperative but continue to be farmed by their owners	22	23
Sale or leasing of plots of land to non-members	25	26
Plots dedicated to non-agricultural uses	6	6
Others	6	6

The answers to the product innovation section, in Table 4, show that 33% of cooperatives surveyed have not promoted new varieties, new crops or cropping systems, or differentiated payment for quality in the last five years, but 25% have somewhat promoted them. Moreover, 52% of them have not promoted organic farming or waste farming in the previous five years, and 75% have not done it for new processed, 4th^,^ or 5th range products in the previous five years.

**Table 4:** Product innovation.

	Frequency	%
*Scale of promotion of new varieties, new crops or cropping systems or differentiated payment for quality in the last 5 years*		
Not at all	35	33
Very little	19	18
Somewhat	27	25
Quite a lot	16	15
A lot	9	8

*Scale of promotion of organic farming or zero waste farming in the last 5 years*		
Not at all	55	52
Very little	10	9
Somewhat	10	9
Quite a lot	16	15
A lot	15	14

*Scale of promotion of new processed, 4th or 5th range products in the last 5 years*		
Not at all	80	75
Very little	10	9
Somewhat	9	8
Quite a lot	4	4
A lot	3	3

The governance innovation section, whose answers are in Table 5, shows that 53% of the agri-food cooperatives surveyed have not promoted women or young people as members in the last five years. Of those who have promoted them, 42% have used the measure of access to operational plans or other aids for their inclusion. In addition to this, 56% of the cooperatives have promoted young people, women, or non-members of the board in the Board of Directors of the cooperative in the last five years, and 72% have no terms limit for the members of the Board of Directors in the cooperative's bylaws or have no plan to implement it.

Table 6 shows 34% of agri-food cooperatives have not participated in inter-cooperation, integration, or other formulas in the last five years. 23% have done it in flexible formulas for collaboration with other organizations, same percentage as those who have done it in binding agreements in the form of commercial partnerships. For those who have participated in one of the different cooperation formulas, 54% have experienced with peer entities.

**Table 5:** Governance innovation.

	Frequency	%
*Promotion of women and youth as members in the last 5 years*		
Yes	50	47
No	56	53

*If yes, which measures have been adapted for inclusion? (multiple response)*		
Membership fee reduction	8	16
Access to financing	11	22
Access to operational plans or other aids (young farmers…)	21	42
Access to land	1	2
Other	19	38

*Promotion of women, young people or non-member of the board in the Board of Directors in the last 5 years*		
Yes	59	56
No	47	44

*Term limits for the members of the Board of Directors included in your cooperative's bylaws or planning to implement it*		
Yes	30	28
No	76	72

**Table 6:** Cooperation formulas applied.

	Frequency	%
*Participation in inter-cooperation, integration or other formulas in the last five years (multiple responses)*		
Nothing at all	36	34
It has explored integration formulas that have not materialized	14	13
It has participated in flexible formulas for collaboration with other organizations	24	23
It has entered into binding agreements in the form of commercial partnerships	24	23
It has participated in merger or integration processes	19	18

*With what type of organization? (multiple responses)*		
With peer entities	44	54
With second-tier cooperatives and other higher-tier entities	31	38
With non-cooperative companies	11	14

Al least 61% of the cooperatives surveyed have a farming services section, as shown in Table 7, and 71% of those who do not have are not planning to implement it. The main agronomic services offered are pest and disease control (93%), land tillage (80%), pruning and waste management, fertilization, and harvesting (three all with 73%). Additionally, 14% have a joint cropping section that consolidates mainly between 10 and 40 hectares (40%).

**Table 7:** Agronomic services and farming sections offered.

	Frequency	%
*Is there a farming services section?*		
Yes	41	39
No	65	61

*If not, possibility of implementing it?*		
Yes	19	29
No	46	71

*Agronomic services offered in farming service section (multiple responses)*		
Pruning and waste management	30	73
Irrigation	24	59
Fertilization	30	73
Land tillage	33	80
Pest and disease control	38	93
Harvesting	30	73
Grouping of plots of land transferred or leased to facilitate their cultivation by professionals, members, or collaborators	5	12
Integral direct management by the cooperative of plots of land transferred or leased by owners, whether members or non-members	13	32

*Is there a joint cropping section?*		
Yes	15	14
No	91	86

*If yes, how much area is grouped? (ha)*		
0 (recently created)	1	7
Less than 10	3	20
Between 10 and 50	6	40
Between 50 and 100	1	7
More than 100	4	27

## 2 Experimental Design, Materials and Methods

In order to collect empirical data of joint cropland management among agri-food cooperatives in Mediterranean Spanish Regions, a questionnaire was designed, according to a preceding study [9], and a online survey was carried out, thanks to the collaboration with agri-food cooperatives federations of Castilla-La Mancha, Comunitat Valenciana, and Murcia, and two second-degree agri-food cooperatives,Anecoop and Unió Nuts, all of them members of the operational group InnoLand, of the European Innovation Partnership for Agricultural productivity and Sustainability (EIP-AGRI).

The questionnaire content was based on aspects that were found to be relevant for joint cropland management. The main characteristics of agri-food cooperatives sought were socioeconomics information, member features, surface area evolution and land abandonment, product innovation, governance innovation, cooperation formulas, and agronomic and farming services. Characteristics of all questions of the questionnaire are shown in Table 8. It was included mainly closed, semi-open and open questions, some of them multi-response and 5-point Likert scale as well. The survey required an estimated 15–20 min to be completed.

**Table 8:** Questionnaire questions characteristics.

	Type
*Part 1. Main characteristics*	
Region	Semi-open-ended
The three main types of products	Multi-response, semi-open-ended
Average turnover value	Open-ended
Number of members	Open-ended

*Part 2. Members*	
Percentage of members under 40 years old	Open-ended
Percentage of professional members under 40 years old	Open-ended
Percentage of members over 65 years old	Open-ended
Percentage of no-professional members	Open-ended

*Part 3. Surface and abandonment*	
Percentage of the area cultivated abandoned by members in the last 5 years	Open-ended
Average annual rate of land abandonment	Closed-ended
Change in terms of cultivated area in the last five years	Closed-ended
Reasons for land abandonment	Multi-response, semi-open-ended

*Part 4. Product innovation*	
Promotion of new varieties, new crops or cropping systems or differentiated payments for quality	Likert, closed-ended
Type of promotion of new varieties, new crops or cropping systems or differentiated payments for quality	Open-ended
Promotion of organic farming or zero waste farming	Likert, closed-ended
Promotion of new processed products, 4th or 5th range products	Likert, closed-ended

*Part 5. Governance innovation*	
Promotion of women and young people as members	Closed-ended
Measures of promotion of women and young people as members	Multi-response, semi-open-ended
Promotion of women, young people or non-members on the Board of Directors	Closed-ended
Inclusion of term limits for the members of Board of Directors in cooperative's bylaws	Closed-ended

*Part 6. Inter-cooperation and mergers*	
Participation in inter-cooperation, integration, or other cooperation formulas	Multi-response, closed-ended
With which type of organization?	Closed-ended

*Part 7. Services provided and sections*	
Is there a farming services section?	Closed-ended
If not, is it possible to implement it?	Closed-ended
If there is, what agronomic services does it offer?	Multi-response, closed-ended
Is there a joint cropping section?	Closed-ended
If not, is it possible to implement it?	Closed-ended
If there is, how much area is it grouped?	Open-ended

Between July 2020 an February 2021 the questionnaire was hosted online and invitations to answer it ware distributed to CEOs of agri-food cooperatives of Mediterranean Spanish Regions. The survey was closed when no responses were collected for a month. From the 1168 invitations to answer the survey which were sent, 112 were finally answered. Informed consent which include statements about the use of the information for academic purposes, and participation was voluntary was obtained from all participants. The anonymity of participants was ensured in the survey which did not included any personal information. After a screening for missing values and eliminating bias answers, the final sample size was 106 valid responses correctly answered.

## Ethics Statements

We confirm that the participants in the survey have given their informed consent to participate. Participants were ensured that their participation was voluntary and that they could leave or pause the online survey at any point. It was guaranteed that any information provided will be treated confidentially and anonymously.

## CRediT authorship contribution statement

**Consuelo Calafat-Marzal:** Conceptualization, Methodology, Investigation, Data curation, Formal analysis, Validation, Supervision, Project administration, Funding acquisition. **Francesc J. Cervera:** Writing – original draft, Formal analysis, Visualization, Writing – review & editing. **Veronica Piñeiro:** Methodology, Writing – original draft, Investigation, Formal analysis, Data curation, Validation, Writing – review & editing. **Paula Andrea Nieto-Alemán:** Software, Resources.

## Declaration of Competing Interest

The authors declare that they have no known competing financial interests or personal relationships that could have appeared to influence the work reported in this paper.

## References

[1] Colombo S., Perujo-Villanueva M. (2017). Analysis of the spatial relationship between small olive farms to increase their competitiveness through cooperation. Land Use Policy.

[2] Deng X., Xu D., Zeng M., Qi Y. (2019). Does Internet use help reduce rural cropland abandonment? Evidence from China. Land Use Policy.

[3] Lasanta T., Arnáez J., Pascual N., Ruiz-Flaño P., Errea M.P., Lana-Renault N. (2017). Space-time process and drivers of land abandonment in Europe. CATENA.

[4] Ma W., Zhu Z. (2020). A note: reducing cropland abandonment in China – do agricultural cooperatives play a role?. J. Agric. Econ..

[5] Parcerisas L. (2015). Landownership distribution, socio-economic precariousness and empowerment: the role of small peasants in Maresme County (Catalonia, Spain) from 1850 to the 1950s. J. Agrar. Change.

[6] Sikor T., Müller D., Stahl J. (2009). land fragmentation and cropland abandonment in Albania: implications for the roles of state and community in post-socialist land consolidation. World Dev..

[7] Terres J.M., Scacchiafichi L.N., Wania A., Ambar M., Anguiano E., Buckwell A., Coppola A., Gocht A., Källström H.N., Pointereau P., Strijker D., Visek L., Vranken L., Zobena A. (2015). Farmland abandonment in Europe: identification of drivers and indicators, and development of a composite indicator of risk. Land Use Policy.

[8] Tudela-Marco L., Garcia-Alvarez-Coque J.M. (2017). Proceedings of the *XI Congreso de La Asociación Española De Economía Agraria Sistemas Alimentarios y Cambio Global Desde el Mediterráneo Orihuela-Elche, 13-15 Septiembre de 2017*.

[9] Piñeiro V., Martinez-Gomez V., Meliá-Martí E., Garcia-Alvarez-Coque J.M. (2021). Drivers of joint cropland management strategies in agri-food cooperatives. J. Rural Stud..

[10] Cook Michael L., Plunkett Brad (2006). Collective Entrepreneurship: An Emerging Phenomenon in Producer-Owned Organizations. Journal of Agricultural and Applied Economics.

[11] Keenleyside Clunie, Tucker Graham (2010). Farmland Abandonment in the EU: an Assessment of Trends and Prospects. Report prepared for WWF.

